# Meta-analysis reveals variations in microbial communities from diverse stony coral taxa at different geographical distances

**DOI:** 10.3389/fmicb.2023.1087750

**Published:** 2023-07-13

**Authors:** Peng-Tao Pei, Lu Liu, Xiao-Li Jing, Xiao-Lu Liu, Lu-Yang Sun, Chen Gao, Xiao-Han Cui, Jing Wang, Zhong-Lian Ma, Shu-Yue Song, Zhi-Hua Sun, Chang-Yun Wang

**Affiliations:** ^1^Key Laboratory of Marine Drugs, The Ministry of Education of China, Institute of Evolution and Marine Biodiversity, School of Medicine and Pharmacy, Ocean University of China, Qingdao, China; ^2^School of Pharmacy, Fujian Health College, Fuzhou, China; ^3^Single-Cell Center, Chinese Academy of Science Key Laboratory of Biofuels, Shandong Key Laboratory of Energy Genetics, Shandong Energy Institute, Qingdao New Energy Shandong Laboratory, Qingdao Institute of Bioenergy and Bioprocess Technology, Chinese Academy of Sciences, Qingdao, China; ^4^Laboratory for Marine Drugs and Bioproducts, National Laboratory for Marine Science and Technology (Qingdao), Qingdao, China; ^5^University of Chinese Academy of Sciences, Beijing, China; ^6^High Performance Computing and System Simulation Platform, National Laboratory for Marine Science and Technology (Qingdao), Qingdao, China; ^7^Department of Mathematics, Ocean University of China, Qingdao, China

**Keywords:** microbiome, stony coral, large geographical distance, diversity analysis, 16S rRNA gene

## Abstract

Coral-associated microbial communities play a vital role in underpinning the health and resilience of reef ecosystems. Previous studies have demonstrated that the microbial communities of corals are affected by multiple factors, mainly focusing on host species and geolocation. However, up-to-date, insight into how the coral microbiota is structured by vast geographic distance with rich taxa is deficient. In the present study, the coral microbiota in six stony coral species collected from the coastal area of three countries, including United States of America (USA), Australia and Fiji, was used for analysis. It was found that the geographic influence on the coral microbiota was stronger than the coral host influence, even though both were significant. Interestingly, the contribution of the deterministic process to bacterial community composition increased as geographical distance grew. A total of 65 differentially abundant features of functions in coral microbial communities were identified to be associated with three geolocations. While in the same coastal area of USA, the similar relationship of coral microbiota was consistent with the phylogenetic relationship of coral hosts. In contrast to the phylum Proteobacteria, which was most abundant in other coral species in USA, Cyanobacteria was the most abundant phylum in *Orbicella faveolata*. The above findings may help to better understand the multiple natural driving forces shaping the coral microbial community to contribute to defining the healthy baseline of the coral microbiome.

## Introduction

As the most species-rich and abundant biomass marine ecosystem, coral reefs are vital for maintaining ecological balance, developing marine drugs and protecting coastlines (Sharp and Ritchie, 2012; Bourne et al., 2016; Rinkevich, 2021). The symbiont, particularly special microbial communities, was recognized as crucially important in both organism function and ecosystem health (Hernandez-Agreda et al., 2017). Microbial communities participate in the nutrient cycling of corals by producing glucose and taking part in the metabolism of nitrogenous and sulfurous substances and provide important protective services for corals by generating antibiotics and competing with pathogens (Pantos et al., 2015; Ziegler et al., 2017). Coral core microbiome is a stable system across hosts, space and time in the communities (Ainsworth and Gates, 2016; Hernandez-Agreda et al., 2016, 2017). While the overall coral microbiome are influenced by various factors, including coral host and geolocation.

According to previous studies, the coral microbial community composition has been demonstrated to change with coral hosts. Earlier studies showed that the coral host was the strongest driving force for the microbial community structures of six Caribbean corals over place and time (Chu and Vollmer, 2016). Later, host identity was proven to play a dominant role in structuring the microbiome of Caribbean corals, while microbial community dissimilarity increased with geographical distance (Dunphy et al., 2019). Recently, the coral host was also reported as the main driver for structural differences in microbial communities in reef corals of the Malay Peninsula (Kanisan et al., 2022). In addition, a study on *Pocillopora verrucosa* and *Turbinaria peltata*, endemic corals in the South China Sea, showed that both host and geographical factors affect coral microbial communities (Chen et al., 2021).

However, other previous studies suggested that the different viewpoints of coral microbiota vary through geolocation. It was found that the microbial communities of five *Acropora loripes* in the four sites of Davies reef have significant differences (Damjanovic et al., 2020). Even at a small distance, the coral microbiota in *Pocillopora acuta* collected from the offshore islands of Singapore were significantly different (Wainwright et al., 2019). These studies revealed that it is necessary to accumulate comprehensive and in-depth knowledge about the variation pattern of the coral microbiome structured by complex driving forces, especially the global range incorporating diverse coral taxa. There is a high possibility of missing the information on driving influence with broad background based on the findings from individual studies using limited locations and taxa.

To date, research on multiple driving forces in shaping coral microbial communities is still insufficient, especially at large geographical distances. In this study, a meta-analysis of six stony coral species from the Pacific Ocean and the Atlantic Ocean with a distance of more than 2,700 kilometers was used to study the coral microbiota variation associated with coral hosts and a large geographic distance. The similarity relationship of microbial communities in the same sea coastal area was constructed and compared with the phylogenetic relationship of coral hosts. The functional traits of coral microbial communities related to geolocation were also discussed.

## Results

### Sequence information

After a series of processing, a total of 21,997,761 high-quality sequences were obtained from 307 samples from three countries (USA: 24–26°N, 80–82°W, Australia: 23–24°S, 151–152°E, Fiji: 18.13°S, 177.42°E; Table S1 for detailed information): 62 samples of *Montastraea cavernosa*, 25 samples of *Orbicella faveolata*, 128 samples of *Pocillopora damicornis*, 36 samples of *Porites astreoides*, 34 samples of *Porites* and 22 samples of *Pseudodiploria strigosa*. A total of 71,182 amplicon sequence variants (ASV) were obtained by clustering the high-quality sequences using DADA2.

### Structural variation pattern of stony coral microbiota in multiple coral species across a large geographical distance

**Figure 1:**
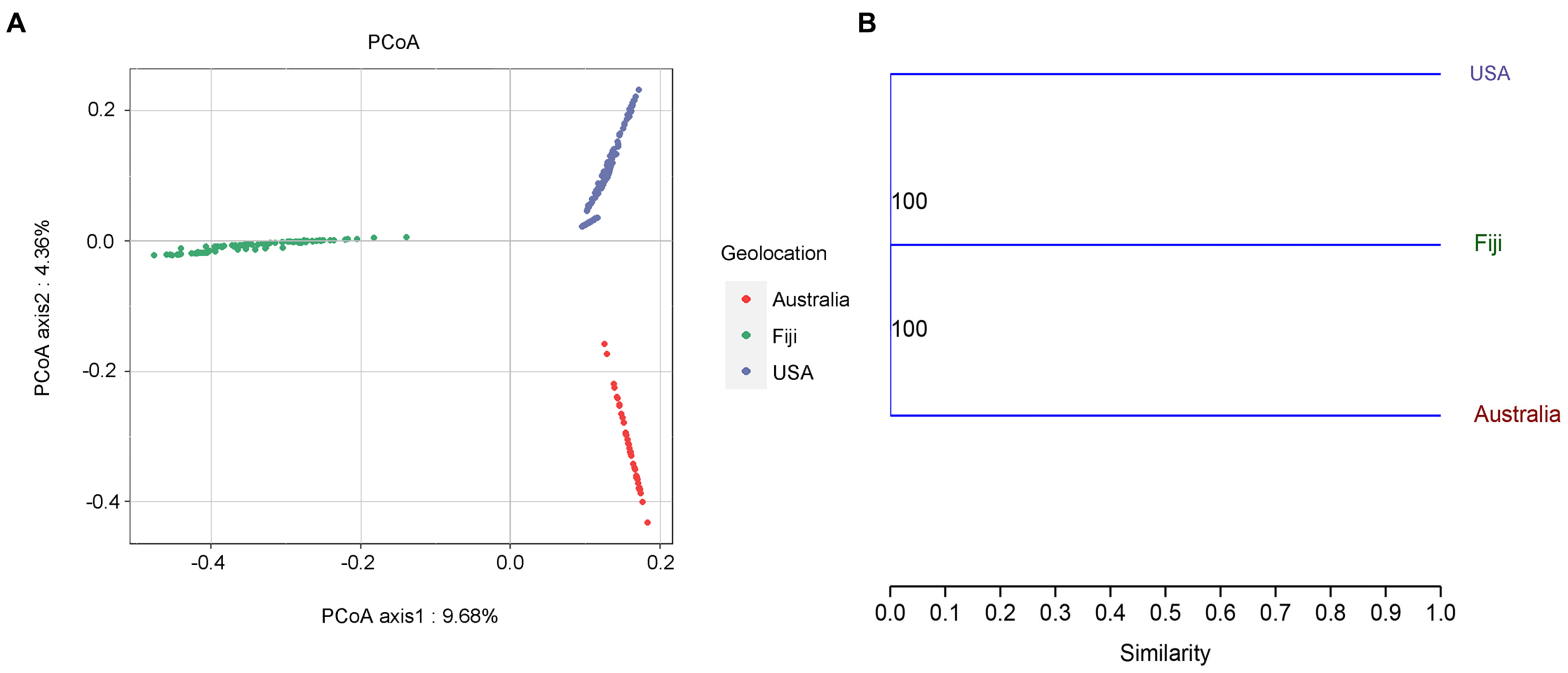
Principal coordinates analysis (PCoA) based on jaccard distance **(A)**. A hierarchical clustering tree of the coral microbial community based on geolocations was constructed by the unweighted pair group method with arithmetic mean (UPGMA). Bootstrap values based on percentage agreement with resampled ASV tables **(B)**.

**Figure 2:**
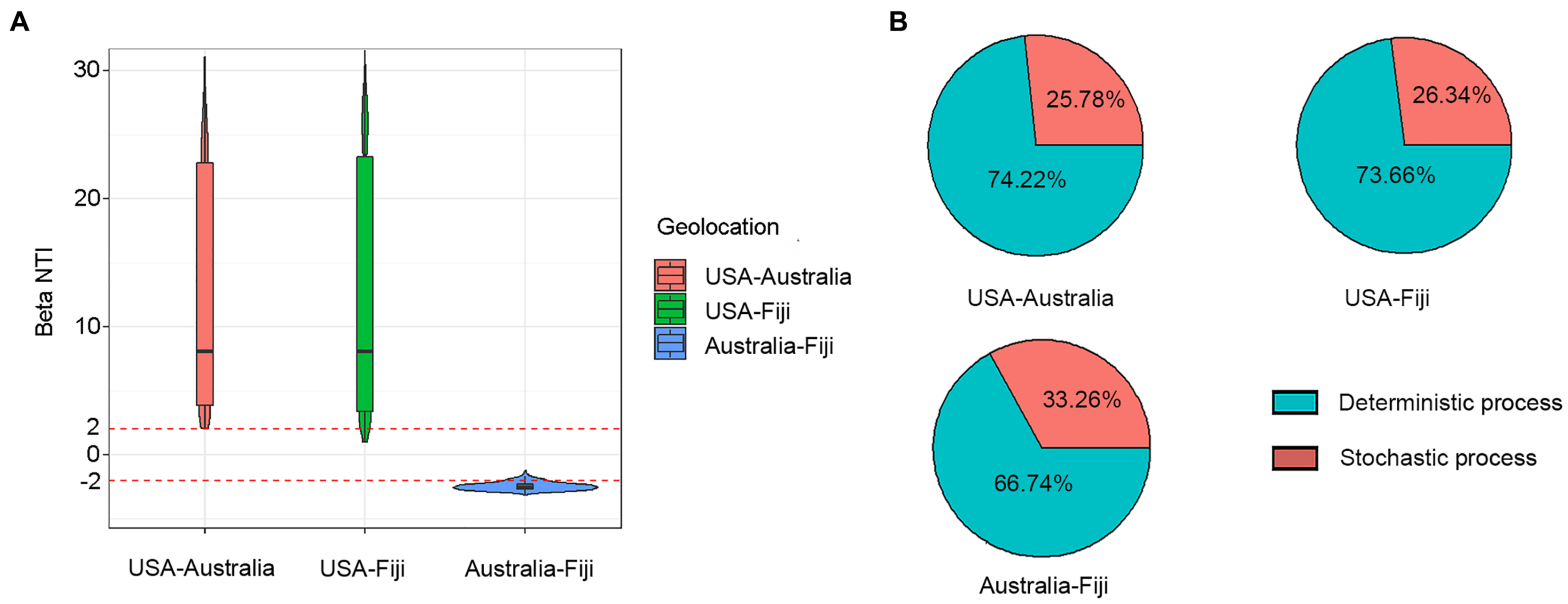
Variation in βNTI median at different geolocations **(A)**. Relative contribution of deterministic and stochastic processes to coral microbial community composition between USA, Australia, and Fiji **(B)**.

To understand the effects on variations in microbial communities from diverse coral taxa across a large geographical distance, the alpha- and beta-diversity of the microbiome were calculated based on the resampled ASV tables. Jaccard distance-based principal coordinate analysis (PCoA) showed that geolocation has a strong and significant impact on the microbial community composition of stony corals (PERMANOVA, permutations = 999, Pseudo-*F* = 21.46, *R^2^* = 0.1238, *p* = 0.001; Figure 1A). Significant differences in microbial community structures were also observed at the coral family and species levels (family level: PERMANOVA, permutations = 999, Pseudo-*F* = 5.701, *R^2^* = 0.0951, *p* = 0.001; species level: PERMANOVA, permutations = 999, Pseudo-*F* = 6.784, *R^2^* = 0.0828, *p* = 0.001). It should be noted that the large geographical distance had a stronger impact on the coral microbiota than the host taxon. The family- and species-level coral hosts had a similar impact degree. Moreover, the hierarchical clustering analysis revealed that all samples were classified into three prominent groups corresponding to the geolocations of Australia, Fiji and USA (Figure 1B). The above results showed that geolocation was the main driving force for the microbial communities of the six coral species across a large geographical distance. Additionally, the β-nearest taxon index (βNTI) analysis revealed that deterministic processes became increasingly influential in shaping the structure of the bacterial community with distance growth (Figure 2).

**Figure 3:**
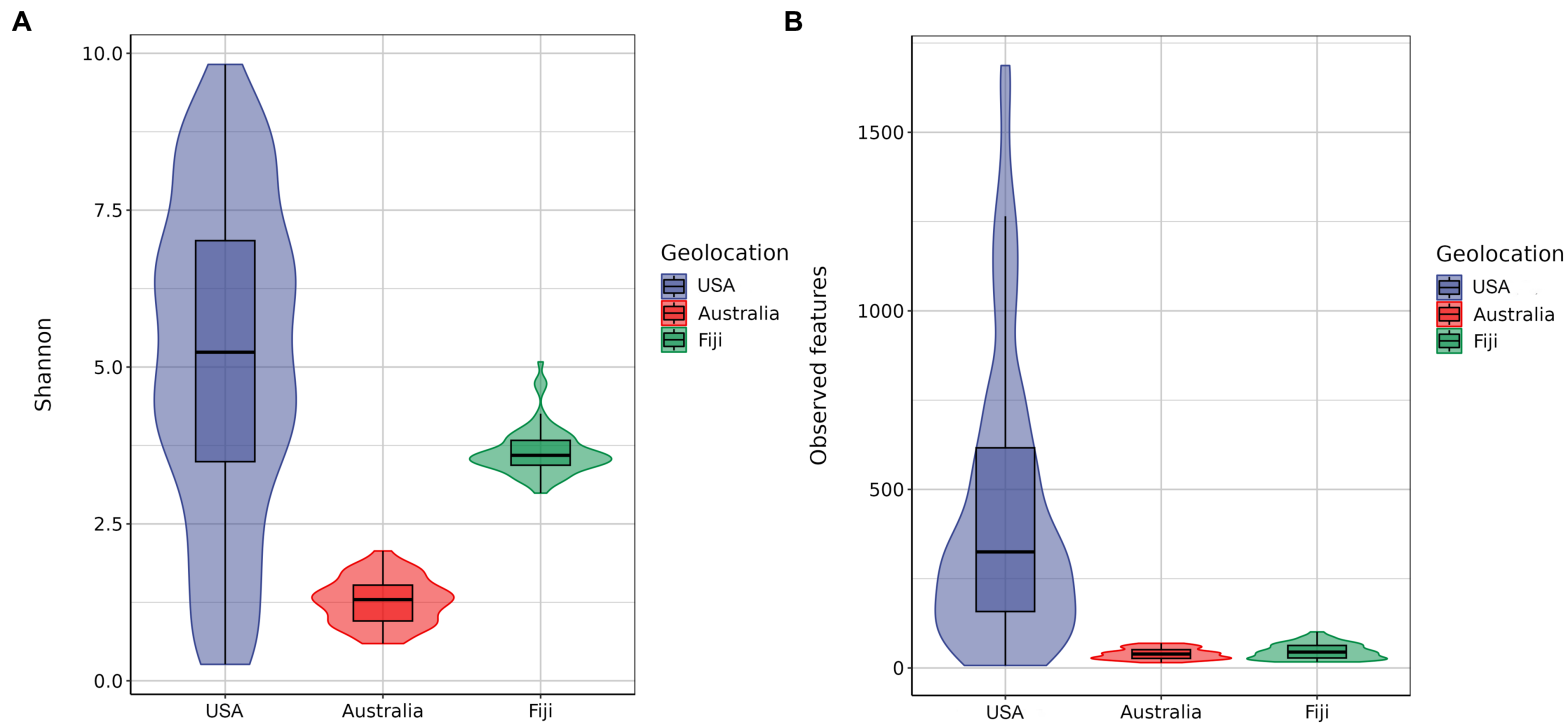
Alpha diversity based on the Shannon diversity index **(A)**. Alpha diversity based on the observed feature diversity index **(B)**.

Furthermore, the Shannon index and observed features index in alpha diversity were calculated, as shown in Figure 3. Kruskal–Wallis pairwise analysis results showed that both Shannon and observed features index (Shannon, mean = 5.1494 ± 2.5049 s.d.; observed features, mean = 448.8101 ± 392.9550 s.d.) of the coral microbiome in the northern subtropical group (USA) was significantly higher than that in the tropical group (Fiji) (Shannon, mean = 3.6470 ± 0.3671 s.d.; observed features, mean = 47.0952 ± 21.1652 s.d.) and southern subtropical group (Australia) (Shannon, mean = 1.2665 ± 0.3655 s.d.; observed features, mean = 40.2045 ± 14.9675 s.d.) (*p* < 0.001). These results suggested that the species diversity, evenness and ASV numbers of the coral microbial communities in the northern subtropical group were significantly higher than those in the tropical and southern subtropical groups, while those in the tropical group were significantly higher than those in the southern subtropical group.

### Structural variation pattern of stony coral microbiota in multiple coral species in the same geographical region

**Figure 4:**
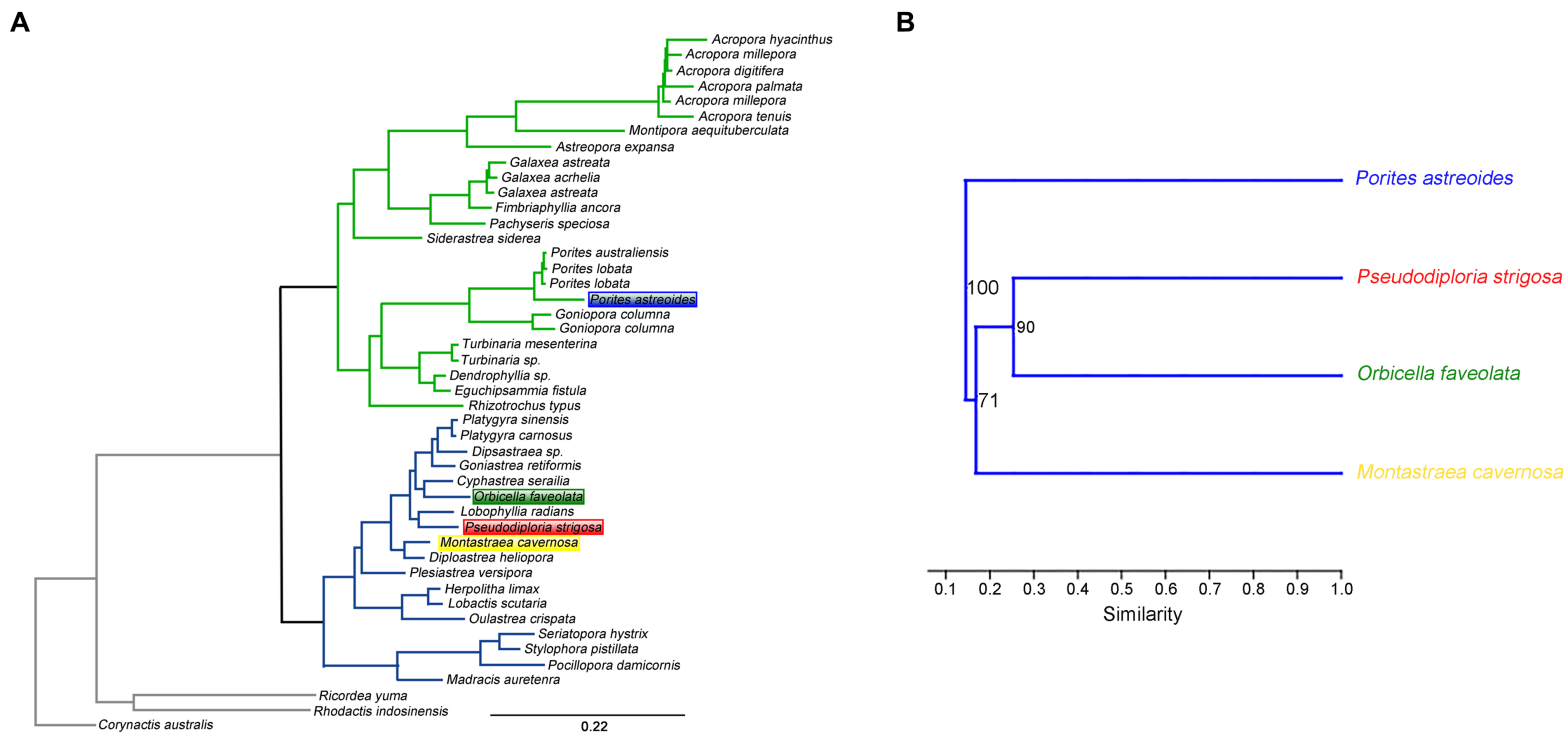
The phylogenetic relationship of stony coral species **(A)**. The similarity of coral microbial community structures constructed by hierarchical clustering **(B)**.

To research the variations in coral microbial communities in the same geographical region, microbiome samples collected from five coral species in the same sea area of USA with a distance of less than 230 kilometers were analyzed. The hierarchical clustering analysis suggested that the similarity of coral microbial community composition could reflect the phylogeny of coral hosts. Except for *P. porites* corals excluded in the phylogenetic tree of a previous report (Quek and Huang, 2019), the similarity relationships of coral microbiota from four coral species were completely consistent with the phylogenetic relationships of coral hosts (Figure 4). The above results revealed that corals with closer phylogenetic relationships could exhibit more similar microbiota compositions in the same geographical region.

**Figure 5:**
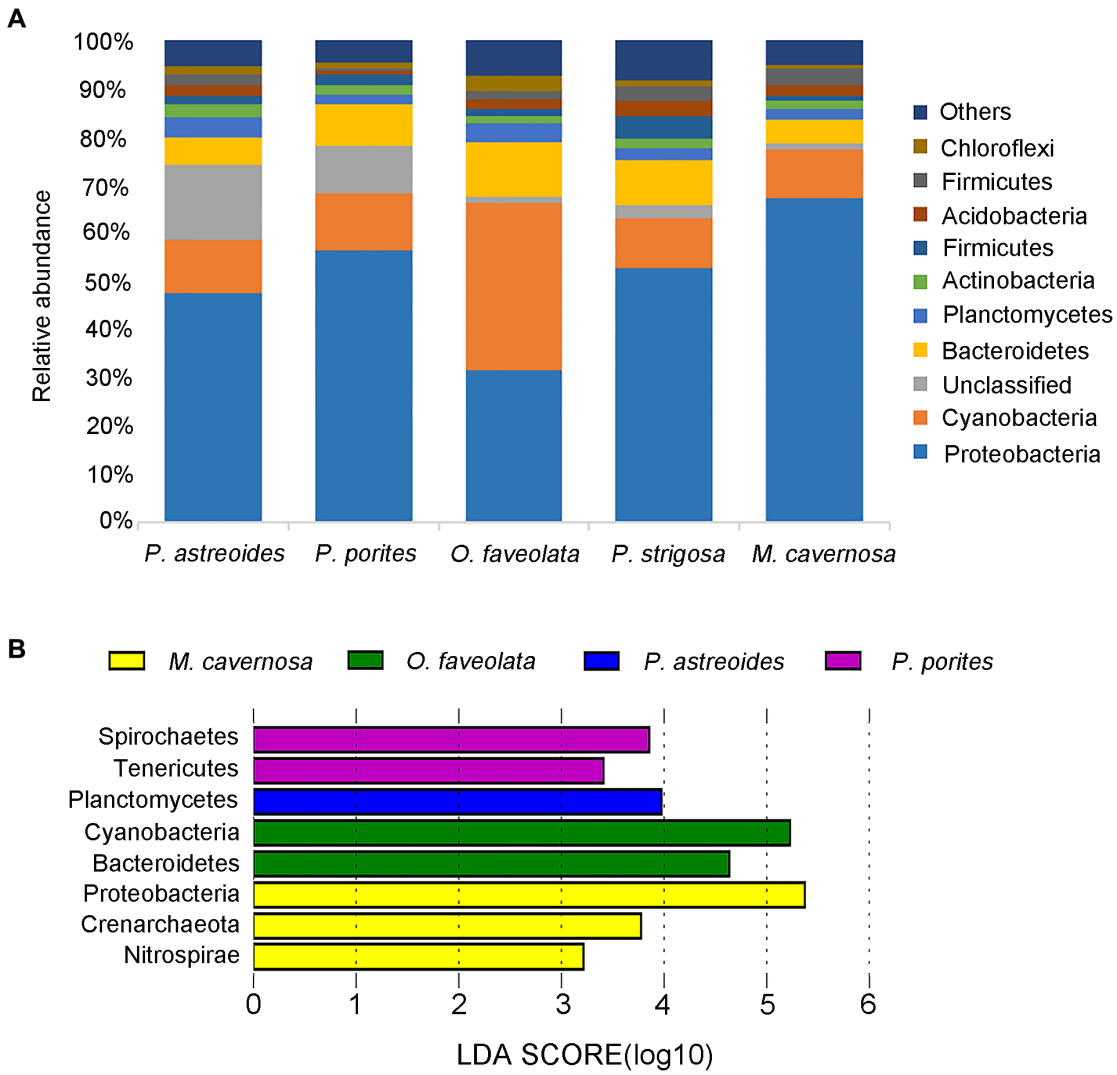
Relative phylum-level abundance of microbial communities for 5 corals **(A)**. LDA value distribution map of different microorganisms. The length of the histogram represents the contribution of different microorganisms (LDA score). The figure shows the microorganisms with significant differences in abundance between different groups when the LDA score was greater than the set value (set to 3). The coral species are highlighted with different colors **(B)**.

Proteobacteria and Cyanobacteria were the dominant phyla in all five coral species, accounting for 52.74 and 14.21% of the relative abundance of total prokaryotes, respectively (Figure 5A). The three most abundant phyla in Porites coral were Proteobacteria, Cyanobacteria and unidentified phyla. The order Rickettsiales was most abundant in *M. cavernosa*, *P. porites* and *P. astreoides*, corresponding to relative abundances of 49.22, 19.08 and 6.17%, respectively, while it was no more than 2.2% in other coral species. Despite permeability to NAD, Rickettsiales was also suggested to be a pathogenic factor that could cause coral diseases in previous studies (Casas et al., 2004; Garcia et al., 2013; Angly et al., 2016; Driscoll et al., 2017; Klinges et al., 2019). Notably, the unidentified phylum was found to account for 12.98% of the total prokaryotic abundance in coral Porites.

The three most abundant phyla in corals *O. faveolata* and *P. strigosa* were Proteobacteria, Bacteroidetes and Cyanobacteria, respectively. It was interesting that the most abundant phylum in coral *O. faveolata* was Cyanobacteria (35.96%), followed by Proteobacteria (32.54%), which was obviously different from the other four coral species. Cyanobacteria were proven to be responsible for nitrogen fixation for corals (Wiebe et al., 1975; Wang et al., 2000; Lesser et al., 2004).

The three most abundant phyla in coral *M. cavernosa* were Proteobacteria, Cyanobacteria and Bacteroidetes. According to the linear discriminant analysis effect size (LEfSe) analysis (Supplementary Table S1), *M. cavernosa* harbored three characteristic bacteria, Proteobacteria, Crenarchaeota and Nitrospirae, at the phylum level compared with the other coral species (Figure 5B). The genus *Nitrosopumilus* belonging to Crenarchaeota was observed to account for 6.68% in coral *M. cavernosa*, while it is almost nonexistent in other corals. *Nitrosopumilus* is an aerobic bacterium, which can efficiently convert ammonia into nitrate (Könneke et al., 2005; Siboni et al., 2008; Jensen et al., 2012; Chekidhenkuzhiyil et al., 2021; Polónia et al., 2021). Nitrospirae is an aerobic bacterium that efficiently converts ammonia into nitrate and is reported to be a common aerobic symbiont in sponges (Hoffmann et al., 2009; Han et al., 2013; Karlińska-Batres and Wörheide, 2013).

### Functional traits of stony coral microbiota in multiple coral species across a large geographical distance

**Figure 6:**
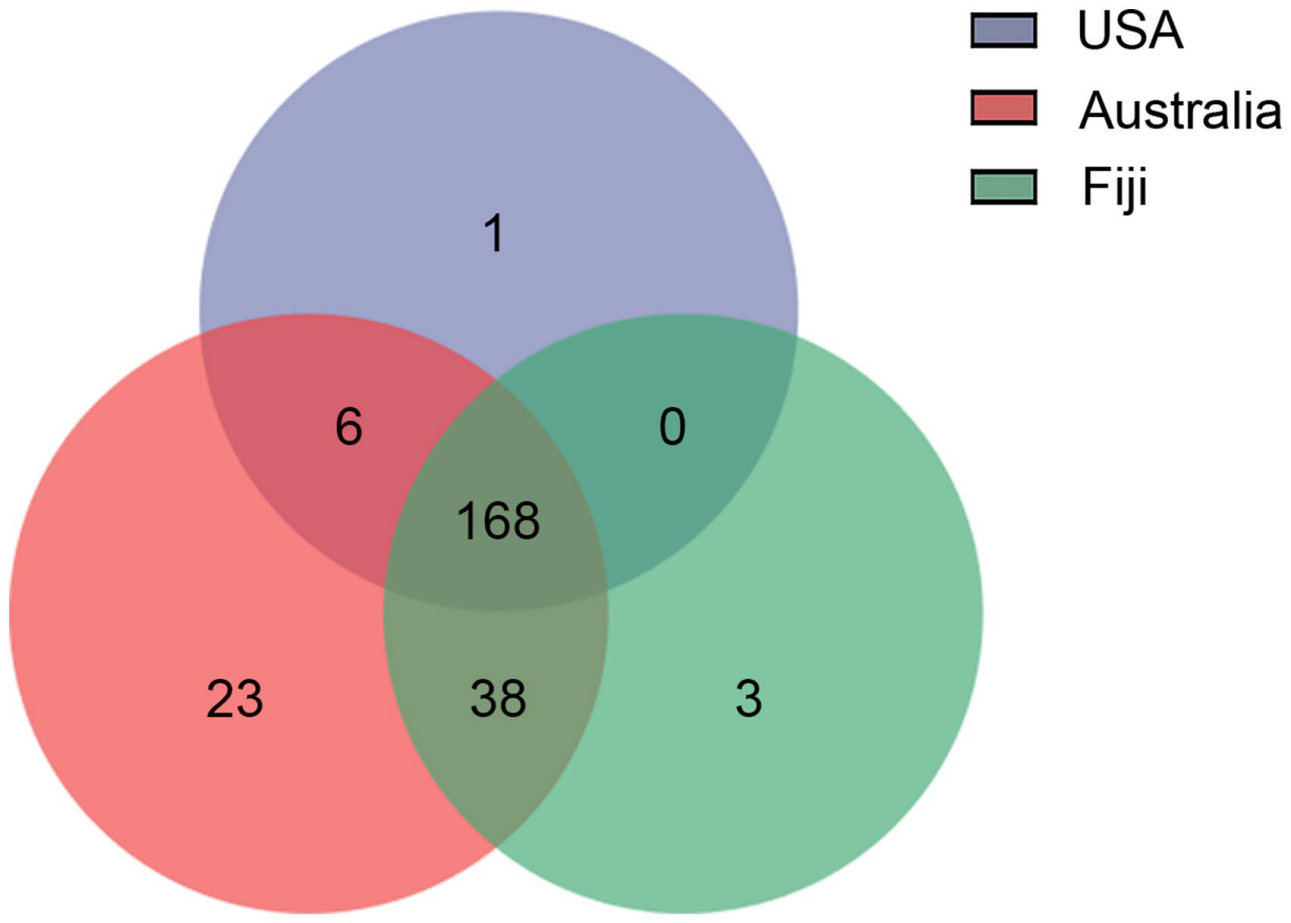
Venn diagram of functional traits of coral microbial communities associated with a large geographical distance.

To further understand the distance-based shifts in microbial communities, the microbial functional traits from stony corals in three countries were elucidated by LEfSe analysis (Supplementary Table S1; Supplementary Figure S1). A total of 65 differentially abundant features of functions in coral microbial communities were identified to be associated with three geolocations. In the southern subtropical group, 37 functional traits, especially several degradation characteristic functions, e.g., degradation of organic acids, nucleosides, acetylene and sucrose, were enriched. In the northern subtropical group, 21 functional traits involved in metabolic pathways, e.g., pentose phosphate pathway and glycolysis, were revealed to be more abundant. In the tropical group, 7 functional traits, such as biotin and organic acid synthesis, were more abundant. In addition, according to the Venn diagram analysis, 168 functions presented in all three groups could be suggested as common functions, accounting for more than 70.29% of all predicted functions (Figure 6). Six functional traits were shared by the northern and southern subtropical groups, including biosynthetic pathways. A total of 38 functional traits were common between the southern subtropical group and tropical group, such as superpathway for organic synthesis. Interestingly, there were no common functional traits between the northern subtropical group and the tropical group. Only 23, 3 and 1 functions were unique to the southern subtropical, northern subtropical and tropical groups, respectively.

## Discussion

Diverse microbial communities play a crucial role in maintaining and shifting the coral holobiont equilibrium. The small conserved coral core microbiome is consistently stable in the communities (Ainsworth and Gates, 2016; Hernandez-Agreda et al., 2016, 2017). However, the overall coral microbiome is dynamic. The detrimental shift in microbial community composition could promote the invasion of opportunistic pathogens and cause coral mortality. Thus, it is necessary to comprehensively and deeply understand the normal variation pattern of the coral microbiome driven by multiple factors to accurately distinguish abnormal changes. Until now, studies researching the composition variation of coral microbial communities have focused on specific geographic regions and coral taxa while ignoring variation patterns by multiple driving forces with various coral taxa from a macro perspective. Previous studies have suggested that both host taxa and geolocation could become the driving forces of coral microbial community structure. However, it presented different conclusions according to the individual studies, even contradictorily (Damjanovic et al., 2020; Kanisan et al., 2022). Recently, it was revealed that microbial community dissimilarity increased with geographical distances of less than 30 kilometers, but the coral host taxon still played a leading role (Dunphy et al., 2019).

In our study, a meta-analysis was used to research the microbiome variation patterns influenced by different geographic distances and diverse host taxa. The results showed that the influence of geolocation factors exceeding the host taxon became the dominant driving force on the coral microbial community at a sufficiently large geographic distance. However, the host taxon was the main influence on the variations in coral microbial community composition in the same coastal area. Moreover, the deterministic process was found to dominate microbial community composition at a larger geographic distance rather than stochastic process, and even increase with distance growth, which was consistent with the previous research (Shi et al., 2018). The result suggested that most ASVs could not interchange between two distinctively environmental condition, leading to predictable differences of their community composition. In contrast, most ASVs could be surviving in the same coastal area where the environmental condition was sufficiently similar. Exploring the variation pattern of a large geographic region with diverse coral taxa would help to provide comprehensive basic knowledge to identify the various healthy states of the coral microbiome.

In addition, the phylum Proteobacteria, as a dominant taxon reported by other studies, was also found to be the most abundant in the coral microbiome of five coral species from USA, Australia and Fiji. Only the phylum Cyanobacteria was the most abundant phylum in the coral *O. faveolata* of USA rather than Proteobacteria (Rubio-Portillo et al., 2018; Wainwright et al., 2019). Cyanobacteria have built coral reefs on Earth for more than three million years. In modern coral reef ecosystems, Cyanobacteria are still ubiquitous, forming a major component and enriching the ecosystem by providing nitrogen through nitrogen fixation. However, Cyanobacteria could also associate with other microbes to play a detrimental role in causing coral tissue lysis and death (Charpy et al., 2012). Coral *O. faveolata,* as a special case, may provide a pathway to promote understanding of the functional evolution mechanism of Cyanobacteria.

It should be pointed out that the structures of coral microbial communities could be affected by diverse factors, including coral hosts, geolocations, coral conditions (health or disease), seasons, sampling methods, and environmental changes (Li et al., 2014; Sharp et al., 2017; Dunphy et al., 2019; Gardner et al., 2019; Vega et al., 2020; Williams et al., 2022). According to the investigation of previous studies for coral microbiome, it was found that the top three most studies are related to coral hosts, geolocations, and coral conditions. In the literature about coral conditions, more than 90% of studies showed that health/disease conditions had a significant effect on the structures of coral microbial communities. Furthermore, the previous research also suggested that 16S rRNA gene amplicon data from different sequencing platforms, and amplified regions could affect resolution and microbial diversity (Schmalenberger et al., 2001; Engelbrektson et al., 2010; Kellogg, 2019). Considering that the metadata about coral hosts, geolocations, coral conditions, sequencing platforms, and amplified regions was provided in most previous studies, yet the metadata about seasons and environmental changes was rarely provided. Therefore, in this study, the filtering conditions of data involved the same sequencing platform, amplified region, and coral condition. After data filtering, the number of coral samples in each geolocation was not exactly the same. In addition, the natural distribution of coral species varied on a large geographical distance. In view of this, the method of PERMANOVA, which is non-parametric, was used for analysis in our study. PERMANOVA is used to compare differences among groups in multivariate datasets without making specific assumptions about the data distribution. Furthermore, we used data from coral adults and larvae of one study (Beatty et al., 2018). Fortunately, it was found that the PERMANOVA statistical results for microbiome of only coral adults were consistent with those of both coral adults and larvae (Supplementary Table S1; Supplementary Figure S2). While our data may not be flawless, the analysis could still provide insights into prevailing trends. In the future, it is necessary to continue efforts to provide detailed information and expand the database of coral microbial communities to identify various patterns of the healthy state. These findings contribute to defining the healthy baseline of the coral microbiome and open the vast possibilities for coral microbiome application in diagnosis and intervention.

## Materials and methods

### Sample data

16S rRNA gene amplicon samples involving six stony coral species collected from three previous publications were merged and reanalyzed (Apprill et al., 2016; Beatty et al., 2018; van Oppen et al., 2018). These original sequence data can be obtained from the NCBI Sequence Read Archive under BioProjects PRJNA324417, PRJNA380169, PRJNA382809, and PRJNA872539 of NCBI and the publication of original papers. The stony coral samples across these four studies to sequence microbial amplicons were taken from coral tissue, mucus and holobiont. The V4 hypervariable region of the 16S rRNA gene was amplified with primers, and then the samples were sequenced on the Illumina platform (Supplementary Table S1).

### Bioinformatics analysis

For the sequences converted to fastq format, Trim Galore v.0.6.6^1^ software was used to filter the sequences with low scores and short lengths maintaining a Phred score threshold of 20 and a length threshold of 100 bp. The paired sequences after quality control were combined using FLASH v.1.2.11 (Magoč and Salzberg, 2011) with a merge rate of more than 80%, then the abiotic sequences were removed with VSEARCH v.2.7.0 (Rognes et al., 2016). FastQC v.0.11.9^2^ software was used to evaluate the quality of data, and unqualified samples were removed. Later, samples with sequencing depth less than 5,000 were filtered. Downstream bioinformatics analysis was performed using QIIME2 v.2020.11.1 (Bolyen et al., 2019). High-quality sequences were clustered into ASV, and Greengenes release 13_8 was used to annotate the sequence as the reference database (DeSantis et al., 2006; McDonald et al., 2012).

### Statistical analysis

Prior to calculating the diversity index, samples were randomly rarefied to the minimum library size (5,800 sequences) with QIIME2 v.2020.11.1 due to large differences in library sizes between different samples. Alpha diversity metrics, including the Shannon index and observed features index, were calculated using QIIME2 v.2020.11.1. The mean value and standard deviation of each metric group were calculated by SPSS v.25 software. Differences in beta diversity were computed using Jaccard dissimilarity matrices and tested via permutational multivariate analysis of variance (PERMANOVA). PERMANOVA analysis was performed in R v.4.2.1 using the vegan package. Variation in community composition among samples was visualized with PCoA. Hierarchical clustering analysis was obtained by using the unweighted pair group method with arithmetic mean (UPGMA) clustering in PAST v.4.0 software, and bootstrap values were based on percentage agreement with the resampled ASV table. All βNTI values were conducted using Picante in R v.4.2.1. If βNTI >2 or βNTI < − 2, deterministic processes were important in shaping the community composition, whereas if values were between −2 < βNTI <2, stochastic processes would play an important role. The zero model was used to quantify the deviation from absolute phylogenetic distance to stochastic phylogenetic distance in this method. The greater the degree of deviation, the more the community was affected by deterministic factors, and the smaller the degree of deviation, the more the community was affected by random factors (Kembel et al., 2010). The phylogenetic tree in Figure 4A was constructed with RAxML v.8.2.11 by Quek in previous study (Stamatakis, 2014; Quek and Huang, 2019).

### Functional profiling based on the microbial community

To better understand the potential functional profiles of the specific microbial taxa in reef habitats, Phylogenetic Investigation of Communities by Reconstruction of Unobserved States (PICRUSt) was applied (Douglas et al., 2020). PICRUSt2 can predict metagenomic functional composition from 16S rRNA gene using marker gene data and a reference genome database. The ASV table of microbial communities with large geographical distances was imported into the “Picrust2_pipelines.py” script in QIIME2 format. The enzyme commission (EC) metagenomes, KEGG orthology (KO) metagenomes and MetaCyc pathway abundances were predicted. The weighted nearest sequenced taxon index (NSTI) score for each sample was calculated (mean NSTI = 0.29 ± 0.16 s.d.). The LEfSe method was used to further analyze the metagenomic prediction count table to identify significantly different functional traits of microbial communities across a large geographical distance [Linear discriminant analysis (LDA) > 3.0 for individual KOs].^3^ A Venn diagram was drawn to highlight shared functions among the different geolocations.

## Data availability statement

The original contributions presented in the study are included in the article/Supplementary material, further inquiries can be directed to the corresponding authors.

## Author contributions

C-YW and LL conceived the study. P-TP, X-LL, CG, Z-LM, S-YS, X-LJ, and Z-HS collected the samples. P-TP, LL, X-LL, X-HC, and JW analyzed the data and prepared the figures and tables. C-YW, LL, P-TP, and L-YS wrote the manuscript. All authors commented and contributed to improving the manuscript and preparing the manuscript for submission, read and approved the final manuscript.

## Funding

This work was supported by the Key Program of National Natural Science Foundation of China (No. 41830535), the Shandong Natural Science Foundation, China (ZR2019BC087), the Major Project of Qingdao Marine Science and Technology Center (2022QNLM030003-1), the Program of Open Studio for Druggability Research of Marine Natural Products, National Laboratory for Marine Science and Technology (Qingdao, China) Directed by Kai-Xian Chen and Yue-Wei Guo, and the Taishan Scholars Program, China.

## Conflict of interest

The authors declare that the research was conducted in the absence of any commercial or financial relationships that could be construed as a potential conflict of interest.

## Publisher’s note

All claims expressed in this article are solely those of the authors and do not necessarily represent those of their affiliated organizations, or those of the publisher, the editors and the reviewers. Any product that may be evaluated in this article, or claim that may be made by its manufacturer, is not guaranteed or endorsed by the publisher.

## Footnotes

^1^https://github.com/FelixKrueger/TrimGalore

^2^https://www.bioinformatics.babraham.ac.uk/projects/fastqc

^3^https://cloud.oebiotech.cn/task/detail/LEfSe-oehw/
