# Supplementary material for: Meta-analysis reveals variations in microbial communities from diverse stony coral taxa at different geographical distances
Source: Front Microbiol. 2023 Jul 13;14:1087750. doi: 10.3389/fmicb.2023.1087750 (PMC10374221; doi:10.3389/fmicb.2023.1087750)
Supplement: Supplementary file 2 [file Data_Sheet_1.PDF]

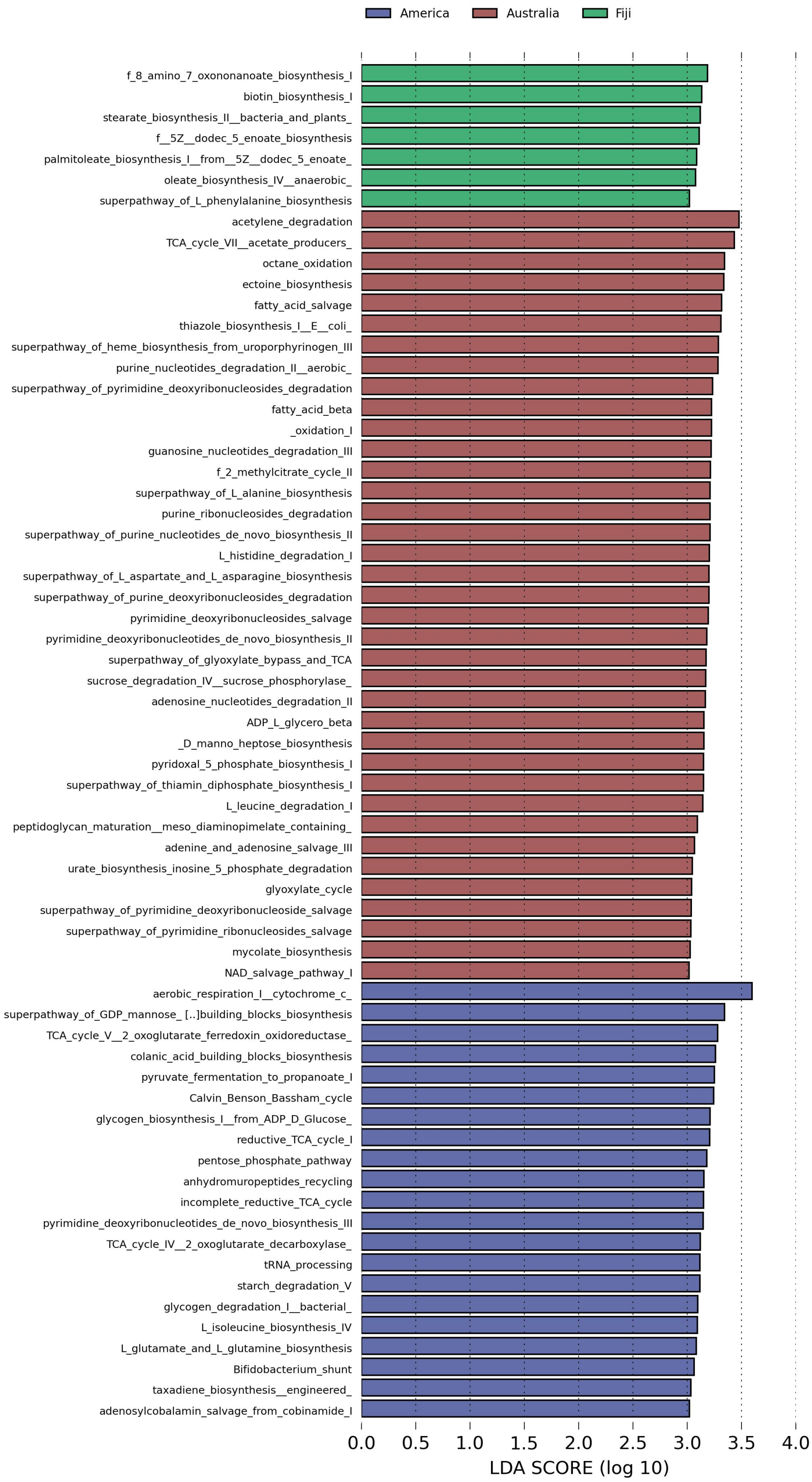

Fig. S1 LDA value distribution map of different functions. The length of the histogram represents the contribution of different functions (LDA score). The figure shows the function of significant differences in abundance between different groups when the LDA score is greater than the set value (set to 3). The coral species are highlighted with different colors.
