# Supplementary material for: Meta-analysis reveals variations in microbial communities from diverse stony coral taxa at different geographical distances
Source: Front Microbiol. 2023 Jul 13;14:1087750. doi: 10.3389/fmicb.2023.1087750 (PMC10374221; doi:10.3389/fmicb.2023.1087750)
Supplement: Supplementary file 3 [file Data_Sheet_2.PDF]

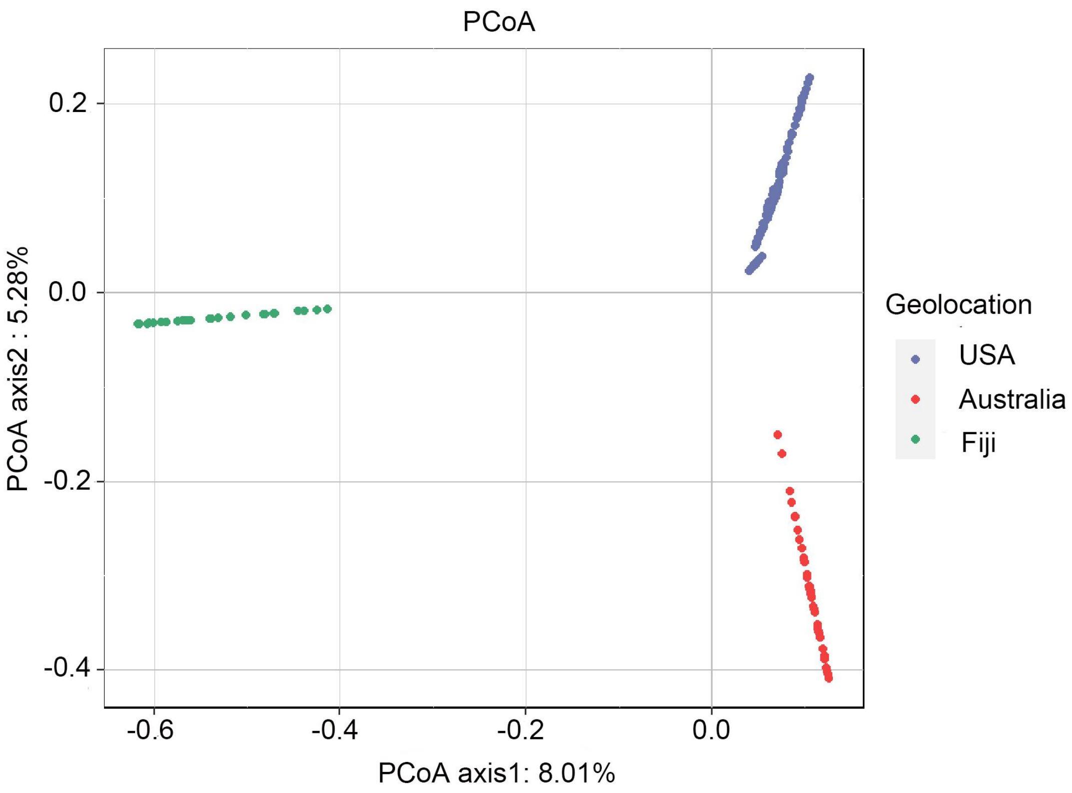

Fig. S2 Principal coordinates analysis (PCoA) based on jaccard distance of coral microbiome from USA, Australia and Fiji (only coral adults).
